# Supplementary material for: From colon wall to tumor niche: Unraveling the microbiome’s role in colorectal cancer progression
Source: PLoS One. 2024 Oct 22;19(10):e0311233. doi: 10.1371/journal.pone.0311233 (PMC11495602; doi:10.1371/journal.pone.0311233)
Supplement: Attachment — Submitted filename: Rebuttal letter.pdf [file pone.0311233.s001.pdf]

CAL POLY

Raul J. Cano, Ph.D.
Biological Sciences Department
California Polytechnic State University
San Luis Obispo, CA 93407
Email: rcano@calpoly.edu

8/21/24

Re: Rebuttal Letter

PLOS ONE Editorial Board

We appreciate the thorough and diligent review of our manuscript, PONE-D-24-11510. We are grateful for the opportunity to revise the manuscript in accordance with the reviewers' comments and the journal's requirements. We believe we have addressed all the concerns raised. Below is a detailed explanation of the revisions made:

Manuscript Formatting: We have made every effort to ensure that the manuscript conforms to PLOS ONE's style requirements, including the proper naming of files.

1. **Data Availability Statement:** We have completed the "Data Availability Statement" on the submission form. Additionally, we have deposited all SRA metagenomic sequences in the NCBI database, under BioProject ID PRJNA 1150116, to be released upon acceptance of the manuscript.
2. **References Section:** The references section has been reviewed and reformatted according to the PLOS ONE style template using EndNote21. The references are now numbered in brackets.
3. **Results and Discussion:** In response to the feedback, we have merged the separate sections on Results and Discussion into a single section titled "Results and Discussion," where the results are discussed immediately after they are presented.
4. **Response to Reviewer Comments:**
    a. **Reviewer #1:** We appreciate the positive feedback and have ensured that all the data available from the study are included in the body of the manuscript. Additionally, we have deposited all the SRA paired end reads, from which all the microbiome analyses were based, in the NCBI SRA database with the BioProject ID number PRJNA 1150116.
    b. **Reviewer #2:** We have formalized the introduction section as suggested. Additionally, we expanded the Methods and Results sections to improve comprehensiveness, ensuring that all critical details and explanations are thoroughly covered. Specifically, we have revised the descriptions of 16S metagenomic sequencing under Microbiome Analysis and the Classification of microsatellite instability based on MMR detection to include more detailed explanations.

We trust that these revisions meet the expectations of the reviewers and the journal. Thank you for considering our revised manuscript. We look forward to your feedback.

Dear Editor:

Sincerely,

Raul J. Cano, PhD
Professor Emeritus
